# Cytotoxic effects of oosporein isolated from endophytic fungus *Cochliobolus kusanoi*

**DOI:** 10.3389/fmicb.2015.00870

**Published:** 2015-09-01

**Authors:** Alurappa Ramesha, M. Venkataramana, Dhamodaran Nirmaladevi, Vijai K. Gupta, S. Chandranayaka, Chowdappa Srinivas

**Affiliations:** ^1^Department of Microbiology and Biotechnology, Bangalore UniversityBangalore, India; ^2^Toxicology and Immunology Division, DRDO-BU-Center for Life Science, Bharathiar UniversityCoimbatore, India; ^3^Discipline of Biochemistry, School of Natural Sciences, National University of Ireland GalwayGalway, Ireland; ^4^Department of studies in Biotechnology, University of MysoreMysore, India

**Keywords:** oosporein, toxicity, ROS, oxidative stress, realtime-Q-PCR

## Abstract

In the present study, oosporein, a fungal toxic secondary metabolite known to be a toxic agent causing chronic disorders in animals, was isolated from fungus *Cochliobolus kusanoi* of *Nerium oleander* L. Toxic effects of oosporein and the possible mechanisms of cytotoxicity as well as the role of oxidative stress in cytotoxicity to Madin-Darby canine kidney kidney cells and RAW 264.7 splene cells were evaluated *in vitro*. Also to know the possible *in vivo* toxic effects of oosporein on kidney and spleen, Balb/C mouse were treated with different concentrations of oosporein ranging from 20 to 200 μM). After 24 h of exposure histopathological observations were made to know the effects of oosporein on target organs. Oosporein induced elevated levels of reactive oxygen species (ROS) generation and high levels of malondialdehyde, loss of mitochondrial membrane potential, induced glutathione hydroxylase (GSH) production was observed in a dose depended manner. Effects oosporein on chromosomal DNA damage was assessed by Comet assay, and increase in DNA damage were observed in both the studied cell lines by increasing the oosporein concentration. Further, oosporein treatment to studied cell lines indicated significant suppression of oxidative stress related gene (*Superoxide dismutase1* and *Catalase* ) expression, and increased levels of mRNA expression in apoptosis or oxidative stress inducing genes *HSP70, Caspase3, Caspase6*, and *Caspase9* as measured by quantitative real time-PCR assay. Histopathological examination of oosporein treated mouse kidney and splenocytes further revealed that, oosporein treated target mouse tissues were significantly damaged with that of untreated sam control mice and these effects were in directly proportional to the the toxin dose. Results of the present study reveals that, ROS is the principle event prompting increased oosporein toxicity in studied *in vivio* and *in vitro* animal models. The high previlance of these fungi in temperate climates further warrants the need of safe food grain storage and processing practices to control the toxic effects of oosporein to humans and live stock.

## Introduction

Endophytic fungi are well known producers of several secondary metabolites including antibiotics, mycotoxins, and volatile organic compounds. Mycotoxins are one of the significant categories of naturally available fungal toxic secondary metabolites produced by several species of fungi with potential toxic effects on human and animal health (Chandra Nayaka et al., 2008, 2009; Venkataramana et al., 2014). These toxins are usual contaminants of cereals and other food grains as well as indoor surfaces, that cause a variety of health effects, ranging from immediate toxic response to potential long-term carcinogenic and teratogenic effects (Chandra Nayaka et al., 2011). The common symptoms, appearing on exposure to mycotoxins include dermatitis, cold and flu, sore throat, headache, fatig, diarrhea, and impaired or altered immune function, as well as kidney disorders which can lead to death (Chandra Nayaka et al., 2013). Historically, mycotoxins are unrelenting problem to farmers and animal husbandry in developing countries (Zain, 2011; Divakara et al., 2014; Venkataramana et al., 2014).

Oosporein is such class of emerging toxic secondary metabolite produced by several species of fungi and has shown the toxic effects on humans as well as animal health (Cole et al., 1974; Aleo et al., 1991). Structurally, Oosporein is a di-symmetric cyclohexadienedione, that differs from other common mycotoxin structures and its production has been reported from *Chaetomium aureum*, *C. cupreum*, *Beauveria bassiana*, *Tremella fuciformis*, *Phlebia mellea*, *Verticillium psalliotae* and *Oospora colorans* (Taniguchi et al., 1984; Nagaoka et al., 2004; Mao et al., 2010; He et al., 2012; Sahab, 2012). Oosporein occurs naturally worldwide in a variety of food grains intended for human and animal consumption and potentially high concentrations are encountered as contaminants in many important crops (Mao et al., 2010). The main route of animal exposure to oosporein is through ingestion of contaminated food stuff such as maize, wheat, and other cereals (Manning and Wyatt, 1984). Oosporein leads to adverse health effects ranging from acute lesions to chronic nephrites in livestock, poultry, and human (Cole et al., 1974; Brown et al., 1987; Ross et al., 1989).

In the past, efforts have been made to explore the cytotoxicity, antiviral, antibacterial, and antifungal properties of oosporein (Terry et al., 1992; Abendstein and Strasser, 2000; Nagaoka et al., 2004; Favilla et al., 2006; Mao et al., 2010). Preliminary reports based on feeding experiments, described the nephrotoxic potential of Oosporein to Cockerels and broiler chicks (Cole et al., 1974; Brown et al., 1987). Oosporein was also toxic to 1 day old chickens (Manning and Wyatt, 1984). Toxicity studies of oosporein in mice and hamsters indicated an LD_50_ value of 0.5 mg kg^-1^ body weight, when injected intraperitoneally (Wainwright et al., 1986). Oosporein inhibits ATPase activity, but the mechanism has not been studied, it is assumed to be a consequence of membrane disruption, since it alters erythrocyte morphology to promote cell lysis (Jeffs and Khachatourians, 1997). Contradictorily few reports have shown no such toxic effect of Oosporein when studied in cells, such as hamster tumor cells, baby hamster kidney cells and invertebrate models like *Artemia salina* and *Daphnia magna* (Abendstein and Strasser, 2000; Favilla et al., 2006). Aleo et al. (1991) studied the nephrotoxic effects of oosporein on rat renal proximal tubules which confered that the proximal tubule viability was altered. However, there was no evidence to support a direct inhibitory effect on mitochondrial respiration at a maximum oosporein concentration of 306 μg mL^-1^. Mao et al. (2010) reported antitumor activity of oosporein on HL-60 and A549 cell lines with an IC_50_ of 28 μM. About cytotoxicity of oosporein, the reports are not consistent and there are no reports on mechanism of oosporein incited cytotoxicity.

Toxicity test are essentially needed to avoid the adverse impacts of these substances on human or animal health, or the environment. Toxicity of the compounds can be studied *in vitro* utilizing auxiliary target organ representing cell lines. In general, mammal origin cells are an attractive to the animal models for investigation of toxicity to humans and farm animals. Therefore, in the present study, we planned to know the mechanism of toxicity of oosporein in Madin-Darby canine kidney (MDCK) and Mouse macrophage (RAW 264.7) cell lines as effects on target organs of mouse including kidney and spleen by using Balb/C mouse model. It was proposed to determine this by measuring the plasma membrane damage, reactive oxygen species (ROS) generation, lipid peroxidation, GSH, mitochondrial membrane potential (MMP), genotoxicity, gene expression of antioxidant, and oxidative stress inducing enzymes. For this study, Oosporein was isolated from endophytic fungus *Cochliobolus kusanoi*, which is associated with *Nerium oleander* under previously described conditions (Alurappa et al., 2014).

## Materials and Methods

### Chemicals

Bovine serum albumin (BSA), minimum essential medium eagle’s (MEM), trypsin (0.1%), MTT [3-(4,5-dimethylthiazol-2-yl)-2,5-diphenyltetrazolium bromide], fetal calf serum (FCS), 2′,7′-DCFH_2_DA, rhodamine 123 were purchased from Sigma Chemical Co. (Saint Louis, MO, USA). Trichloroacetic acid (TCA), Agarose, trypan blue, DMSO, RNA isolation kit, single strand cDNA synthesis kit, and Quiagen fast cyber green master mix were procured from Quiagen-Gamb (Hilden, Germany). All other chemicals and solvents were purchased from Sisco Research Laboratories (Mumbai, India) and Merck (Bangalore, India). The compound oosporein was isolated from the endophytic fungus *C. kusanoi*.

### Cell Culture and Treatments

The MDCK cell line used in the current study was procured from the National Center for Cell Sciences, Pune, India and Mouse macrophage (RAW 264.7) cell line was obtained from ATCC. The cells were equally seeded into plates in 1:1, DMEM/F-12 mixture supplemented with 10% FBS, 2 mM L-glutamine, antibiotic and antimycotic solution (Sigma, St. Louis, MO, USA) in a humid atmosphere of 5% CO_2_ and 95% air at 37°C. To examine possible toxic effects, the cells were treated with oosporein at concentrations ranging from 0.1 to 1 mgmL^-1^ for 24 h.

### Analysis of Toxic Effects using MTT Assay

The effects of oosporein on MDCK and RAW 264.7 cell proliferation were determined by MTT assay (Visconti et al., 1991). Based on the preliminary observation, cells in the exponential phase were seeded onto 96 well plates (5 × 10^4^ cells/well), allowed to adhere for 24 h, and treated with different concentrations (0–200 μM) of oosporein for 3 and 24 h exposure. After the treatment, the medium was removed, cells were washed with PBS and 100 μL of the MTT stock (5 mg/mL) were added to each well. After 4 h of incubation, the solution was removed and 100 μL of DMSO were added to each well. The absorbance was read at 540 nm and cell viability (%) was calculated.

### Lactate Dehydrogenase (LDH) Release Assay

Lactate Dehydrogenase is a marker for cell degeneration. Therefore, the amount of LDH leakage was estimated using LDH-estimation kit (Agappe-11407002) according to the manufacturers’ instructions. In brief, MDCK and RAW 264.7 cells were plated at a density of 5 × 10^4^ cells/well in 24 well plates; after 24 h, the cells were treated with different concentrations (0–200 μM) of oosporein for 6 and 24 h. The cells were precipitated by centrifugation at 2,500 *g* for 5 min at 4°C. The supernatant (100 μL) was mixed with 900 μL of reaction mixture and the percentage release of LDH was assayed.

### Estimation of Intracellular Reactive Oxygen Species (ROS)

The MDCK and RAW 264.7 cells were seeded in 24-well plates at a concentration of 4.0 × 10^5^cells/mL and treated as mentioned earlier with oosporein at concentration range of 0–200 μM. After treatment, the oxidation-sensitive dye DCFH-DA (5 mg/mL) was added to the cells and incubated for 30 min. The cells were then collected after washing twice with PBS and the intracellular ROS formation was detected at an excitation wavelength of 485 nm and an emission wavelength of 535 nm using Hidex plate chameleon^TM^ V (Finland).

### Evaluation of Glutathione hydroxylase (GSH)

The main principle of this method is the reduction of aromatic DTNB to chromogenic thio-nitrobenzoic acid (TNB) by GSH, which is further oxidized to DSSG (disulfide form). Then DSSG is converted back to GSH by glutathione reductase and NADPH. Assessment of the intracellular GSH was carried out by following the method of Griffith (1980). Cells were treated as mentioned earlier and the GSH from the cell free supernatants was quantifed by using GSH estimation kit (Agappe, India).

### Effects of Oosporein on Lipid Peroxidation

The lipid peroxidation was evaluated by measuring malondialdehyde by the method described by Ohkawa et al. (1979) with slight modifications. The MDCK and RAW 264.7 cells were seeded in 75 cm^2^ flask at a concentration of 1.0 × 10^7^cells/mL and incubated at 37°C. After reaching confluency, the cells were treated as mentioned earlier. The cells were harvested, washed with PBS, and sonicated in ice-cold 1.15% KCl with 1% Triton X-100. Then, 100 μL of the cell lysates were mixed with 0.2 mL of 8.1%SDS, 1.5 mL of 20% acetic acid (pH 3.5), and 1.5 mL of 0.8% thiobarbituric acid. The volume was made up to 4.0 mL using distilled water and boiled for 90 min. After cooling, the contents were centrifuged at 1,500 rpm for 10 min, the supernatants were separated, and the absorbance was measured at 532 nm.

### Measurement of Mitochondrial Membrane Potential (MMP)

The toxic effect of oosporein on mitochondrial damage was determined by measuring the MMP using the fluorescent probe rhodamine 123. Cells were cultured in 24 well plates for fluorimetric analysis. After the toxin treatments, rhodamine 123 (10 μg/mL) was added to the cells and incubated for 1 h at 37°C. After washing twice with PBS, the cells were collected and the fluorescence was detected at an excitation wavelength of 485 nm and an emission wavelength of 535 nm using Hidex plate chameleon^TM^ V (Finland).

### Single Cell Gel Electrophoresis (Comet Assay)

The alkaline comet assay was performed to measure the DNA damage and evaluate the toxic effect of oosporein on cells leading to apoptosis. Exponentially growing cells were treated with different concentrations of oosporein (concentration range from 0 to 200 μM) for 2 h. After treatment, the comet slides were prepared briefly, 1 mL aliquots containing 1 × 10^5^ harvested cells were centrifuged and the pellets were re-suspended in 200 μL of 0.75% low melting agarose layered to the frosted slides pre-coated with 1.0% (w/v) normal melting agarose. Finally, a third layer of 0.75% low melting agarose without cells was coated. Subsequently slides were exposed to lysing solution for 1 h at 4°C, rinsed with water and placed in electrophoresis buffer for 20 min and electrophoresis was carried out at 20 V for 20 min. The slides were dipped in neutralization buffer and treated with ethanol for 5 min before staining with 40 μL of ethidium bromide. Flourecence microscope (Olympus, Japan) equipped with Cool SNAP^®^ Pro color digital camera was used to capture the images, and measurements were made by Image Pro^®^ plus software to determine the tail length. Olive tail moment (OTM) was used as the parameter to reflect DNA damage using the formula: %OTM = (head mean) × tail % DNA/100.

### Relative Quantification of Target Gene Expression by Q-RT-PCR

Madin-Darby canine kidney and RAW 264.7 cells were cultured (1 × 10^7^) in 75 cm^2^ flasks and treated with 25–100 μM of oosporein for 2 h. Total cellular RNA was isolated using a commercial RNA isolation kit according to the manufacturer’s instructions (Sigma, St Louis, MO, USA). Equal amounts (2 μg) of RNA were primed with oligo (dT) primers and reverse-transcribed using a HS-RT PCR kit (Sigma, St Louis, MO, USA). Amplification of cDNA was performed in a total volume of 20 μL of SYBR Green I Mastermix (Roche Diagnostics, Germany) containing appropriate primers (**Table 1**), using a Roche LightCycler 480. After initial denaturation (95°C for 10 min), 40 PCR cycles were performed using the following conditions: 95°C, 15 s; 60°C, 15 s; and 72°C, 20 s, at the end of PCR reactions, samples were subjected to a temperature ramp (from 70°C to 95°C, 2°C/s) with continuous fluorescence monitoring. For each PCR product, a single narrow peak was obtained by melting curve analysis of the specific temperature. Each sample for targeted gene expression [*Superoxide dismutase1 (SOD1), Catalase (CAT), HSP70, CAS3, CAS6*, and *CAS9*] was assayed in duplicate and the ΔCT method was used to quantify expression levels based on normalization of housekeeping, β-2 myoglobulin gene. The analysis was performed with Light Cycler relative quantification software.

**Table 1:** Sequences of primers used for quantification of gene expression by quantitative Real time-PCR.

Primer name	Sequence (5′–3′)	Gene target	Reference
CAT F	CCTTTCTGTTGAAGATGCGGCG	*Catalase (CAT)*	Hyejin et al. (2012)
CAT R	GGCGGTGAGTGTCAGGATAG		
SOD1 F	AGGCCGTGTGCGTGCTGAAG	*Superoxide dismutase1*	Hyejin et al. (2012)
SOD1 R	CACCTTTGCCCAAGTCATCTGC		
HSP-70 F	ATTGGGTTGCACACCTTCTC	*Heat shock protein-70*	Tine et al. (2010)
HSP-70 R	GGACAAGTGCAATGAGGTC		
CAS-3 F	ATGGGAGCAAGTCAGTGGAC	*Caspase-3*	Yang et al. (2008)
CAS-3 R	CGTACCAGAGCGAGATGACA		
CAS-6 F	CCAGACAGACAAGCTGGACA	*Caspase-6*	Yang et al. (2008)
CAS-6 R	TGTACCAGGAGCCATTCACA		
CAS-9 F	TTCCCAGGTTTTGTCTCCTG	*Caspase-9*	Yang et al. (2008)
CAS-9 R	GGGACTGCAGGTCTTCAGAG		
B2m-F	ACAGGTTGCTCCACAGGTA	β*-2 myoglobulin*	Venkataramana et al. (2014)
B2m-R	GAGTGCAAGAGATTGAAGAG		


### Histopathological Examination of Oosporein on Spleen and Kidney

To study the effect of oosporein on kidney and spleen, experiments were performed on 4 weeks old male white albino Swiss mice (Balb/C) weighing 20.0 ± 2 g. All the animals used in the study were maintained under hygienic laboratory conditions and experiments were performed in compliance with Institutional Animal Ethical Committee (IAEC) permission in Bangalore University, Bangalore, India as per the guidelines of Government of India. They received only water *libitum* and were not fed for 12 h prior the experiment and the animals were distributed into six groups of four individuals each. The animals were oral administered with different concentrations of oosporein (20, 50, 100, 200, and 500 μM) and the animals without fed with oosporein were referred as sam control. After 4 days of oosporein treatment body physiological changes were observed and animals were euthanized under mild anesthesia, kidney, and spleen were collected for gross and histopathological examinations. Organs were washed with PBS and immediately fixed in 10% buffered formalin and dehydrated using gradient series of alcohol (50, 70, 90, and 100%) and embedded in paraffin blocks and 4–5 μm thickness sections were obtained using semi-automated rotary microtome. The sections were stained with hematoxylin-eosin-methylene blue using TST3 tissue stainer and observed under microscope (EVOS XL Cell Imaging System, Life Technologies, Carlsbad, CA, USA) for pathological changes. The pathological changes of kidney were examined for tubular cell swelling, tubular dilatation, interstitial edema, and necrosis. In case of spleen, splenic granulomas, macrophage infiltration, and splenomegaly were observed as pathological damages. Based on pathological observation the damage was graded as none, mild, moderate, and severe extreme.

## Results

### Effect of Oosporein on Cell Proliferation

**FIGURE 1:**
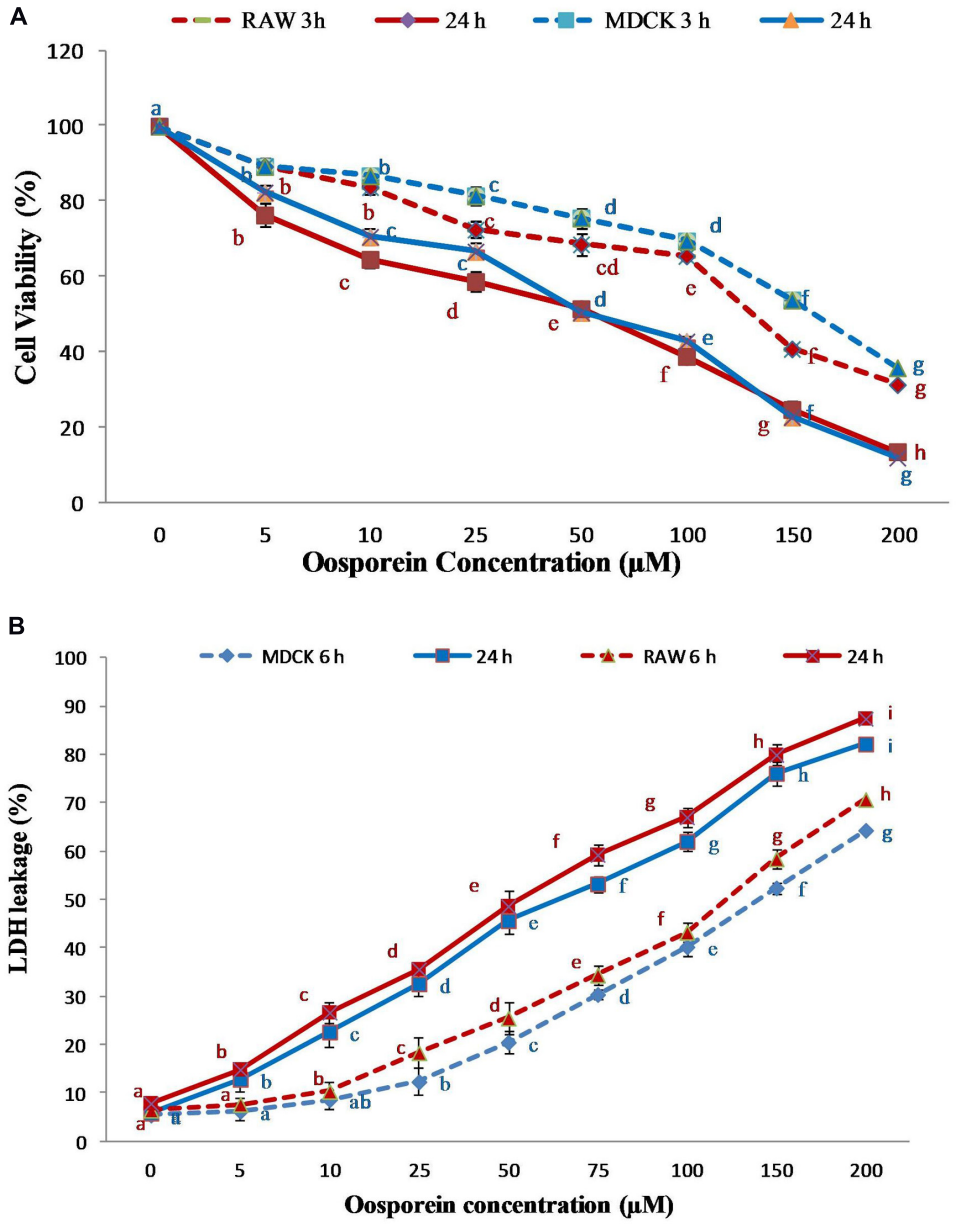
**(A)** Dose dependent cytotoxic effect of oosporein on Madin-Darby canine kidney (MDCK) and RAW 264.7 cell proliferation; **(B)** Dose dependent effect of oosporein on plasma membrane damage in MDCK and RAW 264.7 celllines. Values represent mean ± SD of six parallel experiments. In the figure each series mean values followed by the different letter are significantly different according to DMRT at *p* < 0.05.

The viability of MDCK and RAW 264.7 cells was measured after exposure to oosporein for 3 and 24 h by the method for the determination of the cell numbers by MTT assay. The cell feasibility assessed at 3 and 24 h after the initial introduction of the compound was reduced with increasing concentration. The viability of the cells in control and treated were significantly different in both the exposures. Besides, an increased cell death was observed in oosporein-treated cells as compared to control cells in both the cell lines. The 50% inhibitory concentration (IC_50_) for 24 h was found to be 86 μM for MDCK cells and 78 μM for RAW264.7 cells. Whereas for 3 h exposure time the IC_50_ was found to be at over 150 μM. The higher the oosporein concentration, increase in the cell death rate (**Figure 1A**).

### Effect of Oosporein on Plasma Membrane Damage

The cell death due to plasma membrane damage was evaluated by measuring LDH release. Cells treated with 5–150 μM of oosporein demonstrated increased amounts of LDH leakage, indicating dose dependent toxic effect of oosporein. The results presented in **Figure 1B** show that after treatment for 6 and 24 h, the enzyme leakage was initiated at 25 and 5 μM, respectively, in MDCK cells. The LDH release from RAW 264.7 cells treated with oosporein for 6 and 24 h was observed at 10 and 5 μM, respectively. This indicated that the plasma membrane damage induced by oosporein significantly increased the LDH release rate with increasing concentration of oosporein.

### Estimation of Intracellular Reactive Oxygen Species (ROS)

**FIGURE 2:**
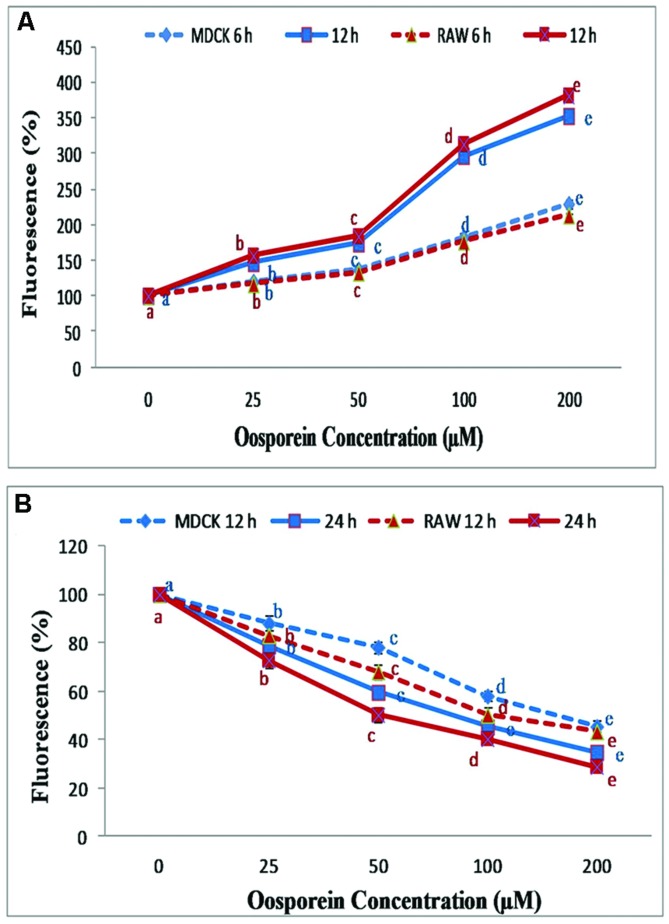
**(A)** Estimation of reactive oxygen species (ROS) production by DCFH-DA using spectrofluorimeter; **(B)** Dose dependent effect of oosporein on Mitochondrial membrane potential (MMP) in MDCK and RAW 264.7 celllines. Values represent mean ± SD of six parallel experiments. In the figure each series mean values followed by the different letter are significantly different according to DMRT at *p* < 0.05.

The intracellular formation of ROS was determined by using the DCFH-DA fluorescence technique. The exposure of cells to oosporein at concentration of 25–200 μM for 6 and 12 h resulted in increased ROS production with increase in concentration. MDCK cells treated with 25, 50, 100, and 200 μM oosporein for 6 h significantly increased ROS production and fluorescence intensity by 120, 140, 200, and 240%, respectively, whereas for 12 h by 150, 190, 300, and 350%, respectively. Increase in ROS production and fluorescence intensity by 120, 138, 190, and 220%, was observed in RAW 264.7 cells treated with various concentrations of oosporein for 6 h, and 155, 185, 318, and 365% for 12 h exposure (**Figure 2A**).

### Measurement of Mitochondrial Membrane Potential (MMP)

To analyze the effect of oosporein on MMP of the cells, the measurement was carried out using rhodamine 123. Here, we observed a decrease in MMP with increasing concentrations 25–200 μM for both time exposure of 12 and 24 h. In MDCK cells, at 12 h exposure to oosporein there was a substantial decrease in fluorescence intensity to the extent of 15, 20, 43, and 58% with increasing concentration of 25, 50, 100, and 200 μM, respectively. Whereas on 24 h exposure of cells to oosporein, fluorescent intensity was decreased to 20, 40, 53, and 63% with increasing concentration. Similar effects were observed in RAW 264.7 cells treated with oosporein. The fluorescence intensity decreased to the extent of 18, 25, 50, and 60% at 12 h exposure of cells to oosporein and on 24 h exposure, the fluorescent intensity was decreased to 25, 50, 60, and 68% with increasing concentration of 25, 50, 100, and 200 μM, respectively (**Figure 2B**).

### Effect of Oosporein on Production of Glutathione Hydroxylase (GSH)

**FIGURE 3:**
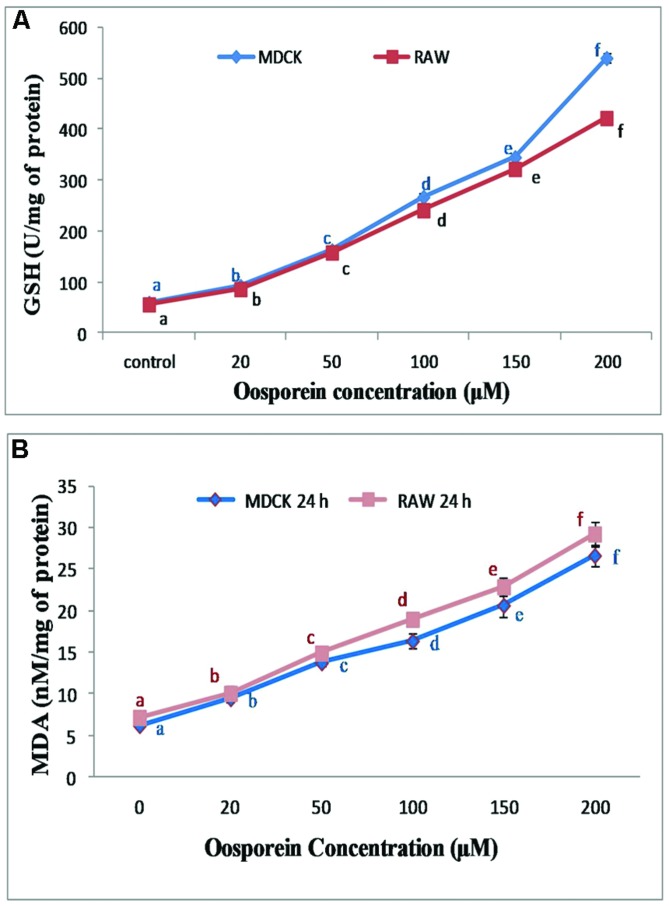
**(A)** Dose dependent effect of oosporein on Glutathione hydroxylase (GSH) in MDCK and RAW 264.7 celllines; **(B)** Estimation of lipid peroxidation products by TBARS assay. Values represent mean ± SD of six parallel experiments. In the figure each a mean values followed by the different letter are significantly different according to DMRT at *p* < 0.05.

The exposure of cells to oosporein at range of concentrations 0–200 μM for 24 h produced an increased concentrations of GSH. MDCK cells treated with 25, 50, 100, 150, and 200 μM concentrations of oosporein significantly increased Glutathione hydrolase production by 90, 160, 230, 350, and 540 U/mg of protein, respectively. The Glutathione hydrolase production in RAW 264.7 cells exposed to various concentration of oosporein was 90, 160, 210, 320, and 410 U/mg of protein (**Figure 3A**).

### Effect of Oosporein on Lipid Peroxidation

Lipid Peroxidation was assessed by measuring malondialdehyde (MDA) formation in cells, which were pre-treated with 20, 50, 100, 150, 200 μM oosporein. The fluorescence intensity increased in the treated cells compared to control cells. There was ∼0.6, ∼1.3, ∼1.6, ∼2.3, ∼3.3, and ∼0.6, ∼1.4, ∼1.8, ∼2.5, ∼3.6-fold increase in fluorescence intensity with increasing concentrations of oosporein in MDCK and RAW 264.7 cells, respectively (**Figure 3B**). This shows that oosporein increased MDA level in cells essentially, which is a part of oxidative lipid peroxidation.

### Single Cell Gel Electrophoresis (SCGE; Comet Assay)

**FIGURE 4:**
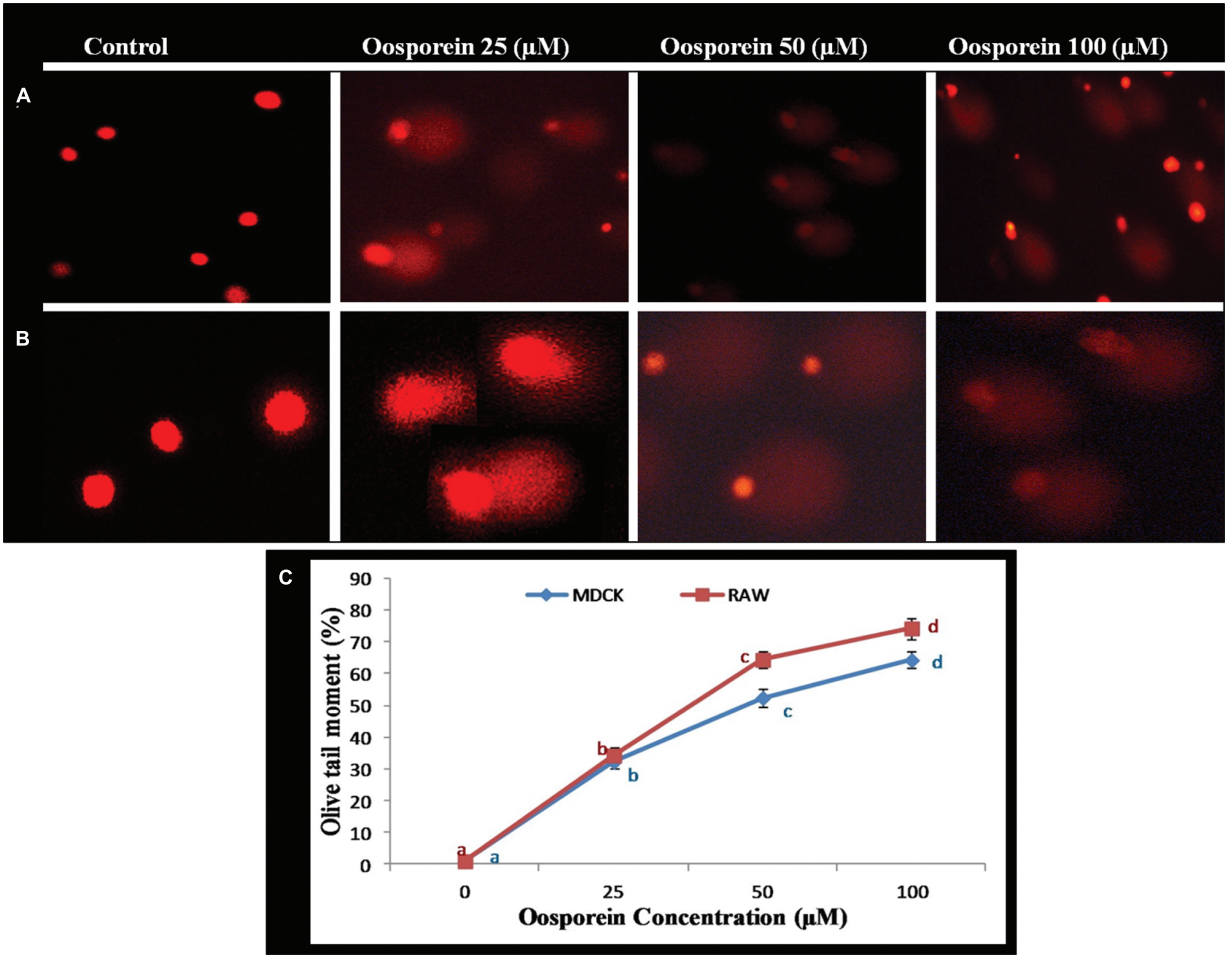
**(A)** Estimation of DNA damage induced by oosporein in MDCK celllines. **(B)** Estimation of DNA damage induced by oosporein in RAW 264.7 celllines. **(C)** Effect of different concentrations of oosporein on nuclear organization of MDCK and RAW 264.7 cells. Values represent mean ± SD of six parallel experiments. In the figure each series mean values followed by the different letter are significantly different according to DMRT at *p* < 0.05.

Comet assay was performed to determine the genotoxicity of the substance due to single strand breaks of DNA. To focus the toxic effect of oosporein to induce DNA damage, cells were treated with various concentrations of oosporein for 2 h. The OTM which was used as the parameter to reflect DNA damage increased by∼32, ∼54, and ∼65% for 25, 50, and 100 μM of oosporein concentration, respectively, in MDCK cells (**Figures 4A,C**). In case of RAW 264.7 cells exposed to oosporein the OTM increased by ∼32, ∼68, and ∼75% for 25, 50, and 100 μM, respectively (**Figures 4B,C**). In control cells the tail length was ∼10.0 mm while at 100 μM oosporein it increased up to ∼20 mm (**Figures 4A–C**). The results of this test continuously demonstrate that oosporein caused significant DNA damage.

### Relative Quantification of Target Gene Expression by Q-RT-PCR

#### Effect of Oosporein on SOD and CAT Gene Expression

**FIGURE 5:**
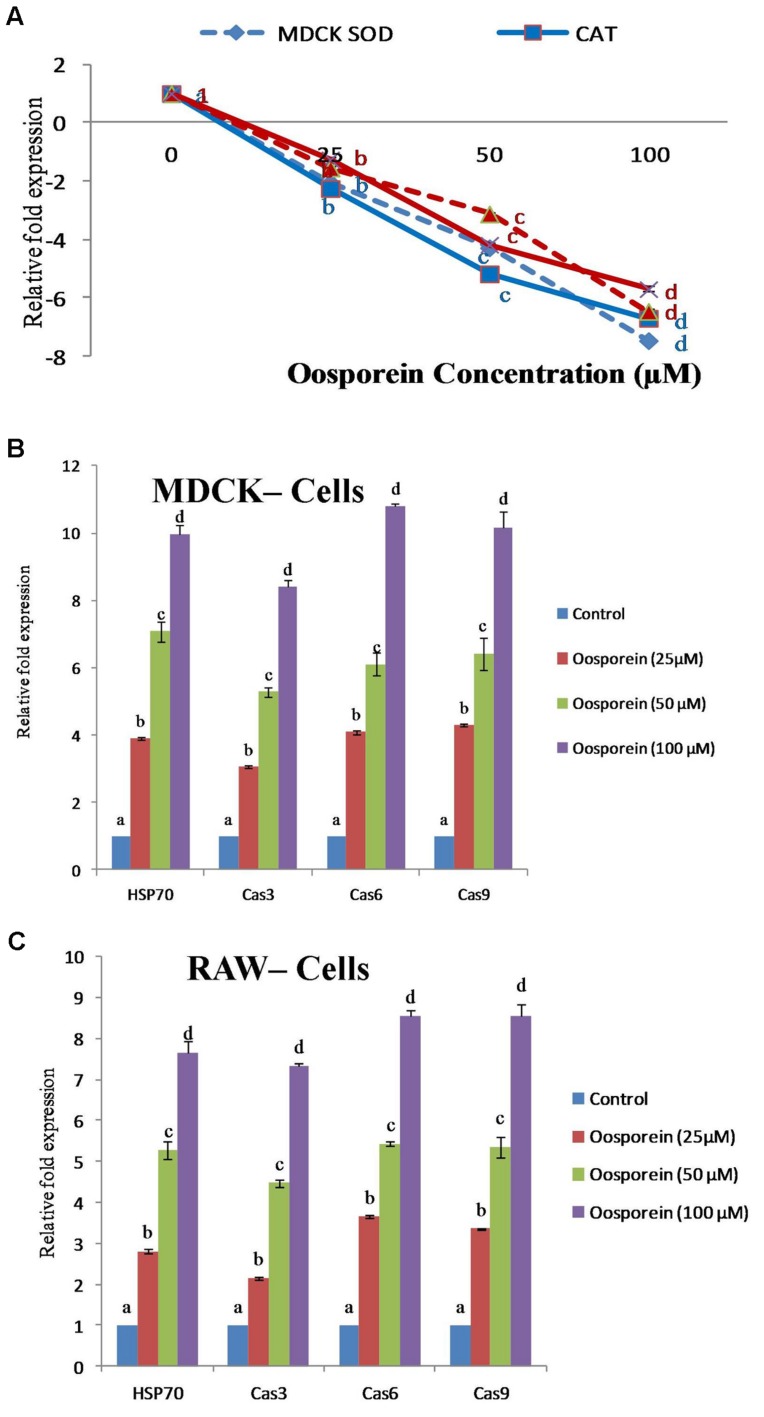
**(A)** Dose dependent effects of oosporein on Oxidative biomarker gene expression of *Superoxide dismutase1 (SOD1)* and *Catalase (CAT)* genes quantified by real time PCR. **(B)** Dose dependent effects of oosporein on apoptosis inducing gene expression of *HSP70*, Cas3, Cas6, and Cas9 genes quantified by real time PCR in MDCK cellline. **(C)** Dose dependent effects of oosporein on apoptosis inducing gene expression of *HSP70*, Cas3, Cas6, and Cas9 genes quantified by real time PCR in RAW 264.7 cellline. Values represent mean ± SD of three parallel experiments. In the figure each series mean values followed by the different letter are significantly different according to DMRT at *p* < 0.05.

To explore whether oosporein intervened on the Oxidative biomarker, gene expression of antioxidant enzymes of *SOD1* and *CAT* in cells were measured. The *CAT* and *SOD1* gene response were assessed by Q-RT-PCR. Compared to control cells, the oosporein treated cells strongly down-regulated *CAT* and *SOD1* mRNA levels with increasing concentration of oosporein (**Figure 5A**). The expression of *CAT* gene decreased by ∼3 to ∼7.5-fold and ∼2.5 to ∼6-fold in MDCK cells and RAW 264.7 cells, respectively, with increasing concentration of oosporein from 25 to 100 μM. There was ∼3 to ∼8.4 and ∼2.8 to ∼6.5-fold decrease in the expression of SOD1 gene in MDCK cells and RAW 264.7 cells, respectively, on exposure to 25–100 μM of oosporein (**Figure 5A**).

#### Effects of Oosporein on *HSP70*, CAS3, CAS6, and CAS9 Gene Expression

Oosporein mediated effect on gene expression of apoptosis or oxidative stress inducing HSP70 (Heat shock protein *70), Cas3 (Caspase 3), Cas6 (Caspase 6), and Cas9 (Caspase 9) gene expression was measured as mentioned earlier.* Expression profile for *HSP70*, Cas3, Cas6, and Cas9 were estimated by quantitative real time RT-PCR. In MDCK cells treated with oosporein at 25–100 μM concentrations up-regulated gene expression of *HSP70*, Cas3, Cas6, and Cas9 mRNA levels in a dose dependent manner was observed. The expression of HSP70 gene increased ∼2.3 to ∼7.8-fold with the increase concentration from 25 to 100 μM of oosporein. The expression of Cas3, Cas6, and Cas9 genes increased ∼2 to ∼7, ∼2.8 to ∼8.6, and ∼3 to ∼7.5-fold, respectively, in cells treated with 25–100 μM of oosporein (**Figure 5B**). Similar response was observed in case of RAW 264.7 cells exposed to various concentrations of oosporein. Gene expression of *HSP70*, Cas3, Cas6, and Cas9 were increased ∼2.5 to ∼6.5, ∼2 to ∼6, ∼3.5 to ∼7.5, and ∼3 to ∼8-fold, respectively (**Figure 5C**).

#### Histopathological Examination

**FIGURE 6:**
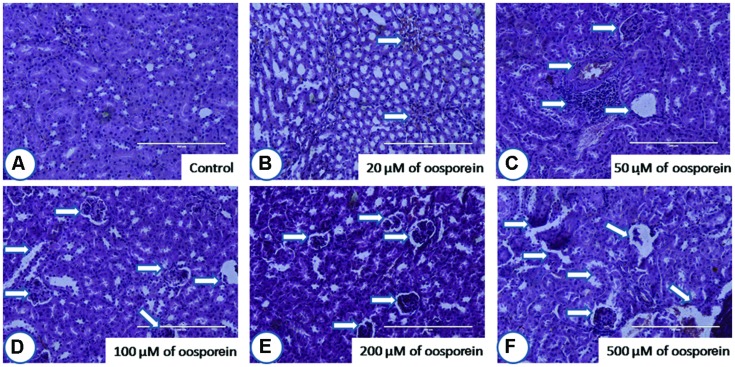
**Histopathological observations of kidney upon treatment with different concentrations of oosporein**. Histopathology of kidney stained with hematoxylin-eosin-methylene blue. **(A)** Control kidney showed no histopathological damage, whereas oosporein exposed kidney showed clear evidence of histopathological damage with cortical tubular dilation with epithelial vacuolation and necrosis as shown as arrow marks. Damage was graded as **(B)** mild, **(C)** mild, **(D)** moderate, **(E)** moderate, and **(F)** severe extreme.

**FIGURE 7:**
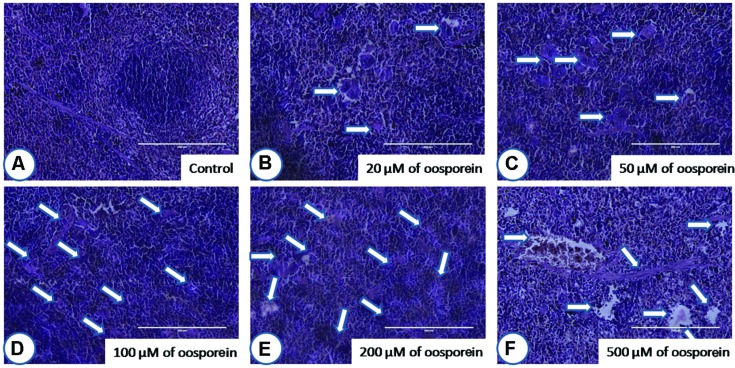
**Histopathological observations of spleen upon treatment with different concentrations of oosporein**. Histopathology of spleen stained with hematoxylin-eosin-methylene blue. **(A)** Control spleen showed no histopathological damage, whereas oosporein exposed spleen showed clear evidence of histopathological damage with splenic granulomas, macrophage infiltration and splenomegaly as shown as arrow marks. Damage was graded as **(B)** mild, **(C)** mild, **(D)** moderate, **(E)** moderate, and **(F)** severe extreme.

After staining the target organ tissue sections, histopathological observations were made with the help of Dr. Nataraj, Assistant Professor, Mysore University. Mouse orally treated with oosporein at a concentration of 20 μM did not show much significant pathological signs in comparison with the untreated sam control mice. On the other hand, 50 μM and above concentrations of oosporein significantly affected both the studied organs architecture. Oosporein effects on kidney were recorded as (shown as arrows on figures) cortical tubular dilation with epithelial vacuolation and necrosis (**Figure 6**) and on spleen were noted as damage in prominent germinal lymphoid centers with splenic granulomas and macrophage infiltration (shown as arrows on figures; **Figure 7**).

## Discussion

The present study was aimed to establish the possible toxic mechanisms of oosporein on *in vitro* and *in vivo* animal models. The mechanism of action of mycotoxins in mammalian systems may be explained by multiple effects on cellular and sub cellular structures (Venkataramana et al., 2014). The *in vitro* cytotoxic effects of oosporein on MDCK and RAW 264.7 cells were measured by MTT assay; cell viability was assessed at 3 and 24 h time intervals. The reduction in cell viability upon oosporein treatment was observed to be dose dependent. The effects of oosporein on plasma membrane leakage were assessed by LDH leakage assay, cells treated with 0–200 μM of oosporein demonstrated that the amount of LDH leakage significantly increased in a dose dependent manner and LDH results are well correlated with the MTT assay. The plasma membrane damage induced was clearly evident by increased LDH release rate with increasing concentrations of oosporein. Strasser et al. (2000) reported the toxic effects of oosporein to animals. In an earlier report, Abendstein and Strasser (2000) demonstrated that oosporein at 600 ng/mL did not show an adverse cytotoxic effect on two different mammalian cell-lines. In addition, Wainwright et al. (1986) reported oosporein at 100 ng/mL had no lethal effect on hamster tumor cells and baby hamster kidney cells. However, our report is supported by the findings of Mao et al. (2010) who reported oosporein at 28 μM level showed anticancer activity on HL-60 and A549 cells.

Reactive Oxygen Species plays an important role in oxidative damage to cellular system which leads to cell injury and death (Ghaffari et al., 2014). In the presence of ROS, DCFH is oxidized to highly fluorescent dichlorofluorescein (DCF; LeBel et al., 1992). Therefore, the intracellular DCF fluorescence was used as an index to quantify the intracellular ROS (Nirmaladevi et al., 2014). In the present study oosporein significantly increased the ROS formation in a dose dependent manner; generation of ROS in turn leads to lipid peroxidation (Venkataramana et al., 2014). Lipid Peroxidation was assessed by measuring the MDA levels. Cells pre-treated with oosporein, significantly increased lipid peroxides, these results were supported by the earlier reports of Aleo et al. (1991). The produced MDA can bind to DNA to generate mutagenic adducts and lead to malfunction of DNA (Chaudhary et al., 1994). These processes have been implicated in the pathogenesis of several systemic diseases including that of kidney (Emin, 2012). The increased GSH level leads to the loss of mitochondrial enzyme activity by producing oxidized glutathione (Benard and Balasubramanian, 1995). In the present study, effect of oosporein on GSH was assessed; MDCK and RAW 264.7 cells treated with different concentrations of oosporein significantly increased GSH production in a dose dependent manner. These findings suggest the role of ROS in oxidative damage of the studied cells.

Further, various ROS and Lipid peroxidation products damage the cell membrane and decrease MMP (Lee et al., 2010). Estimation of MMP collapse is a landmark to evaluate stress induced apoptotic cell death due to mitochondrial membrane damage (Lin et al., 2009). In the present study effect of oosporein on MMP collapse was assessed by fluorescent rhodamine 123. With decrease in MMP the intracellular rhodamine 123 dye subsequently gets released from the plasma membrane and shows decreased fluorescence (Ghaffari et al., 2014). The diffusion and accumulation of rhodamine 123 in mitochondria is directly proportional to the extent of MMP (Ubl et al., 1996). In the present study, we observed a significant decrease in MMP in Oosporein treated cells, indicating depolarization of mitochondrial membrane. Jeffs and Khachatourians (1997) reported oosporein inhibits erythrocyte membrane ATPase activity in a dose-dependent manner. Failure of active transport of ions Ca^2+^ Na^+^, and K^+^ in ATPase pump as a result of a combination of energy deficiency, production of ROS leads to depolarization of mitochondrial membrane (Xue et al., 2003; Jousset et al., 2007). In the present investigation, the decreased MMP may be due to inhibition of Ca^2+^-ATPases and Na^+^/K^+^-ATPase by oosporein leading to mitochondrial membrane depolarization.

Previous study has shown that lipid peroxides are involved in DNA damage by formation of DNA single strand breaks or alkali-labile sites (Venkataramana et al., 2014). SCGE assay was performed to analyze the DNA double strand breaks. The DNA damage was assessed by measuring the tail length of comet (Venkataramana et al., 2014). In the present study oosporein induced DNA damage as observed by the comet assay revealed increased tail length and tail intensity (OTM) with increasing oosporein concentration proving that the compound is genotoxic, and the results are in conformity with the earlier reports (Mally et al., 2005; Leire et al., 2007). Terry et al. (1992) stated that oosporein binds to the DNA binding site of the Herpes simplex type 1 DNA polymerase or intercalates into DNA, thereby disrupting the DNA template.

The oosporein induced ROS generation may be due to destruction of antioxidant enzymes like *SOD1* and *CAT*, which are involved in the cell defense system. The effect of oosporein on gene expression of the antioxidant enzymes *SOD1* and *CAT* were monitored by quantitative real time -PCR. In the present study, we found strongly down-regulated *CAT* and *SOD1* mRNA levels in oosporein treated cells. This reveals that oosporein affects the *CAT* and *SOD1* at transcription level leading to destruction of the cell defense system. Previous report has indicated that after exposure of rats to ochratoxin A, a decrease in the antioxidant activities of *CAT* and *SOD* was observed in the kidney samples (Ozcelik et al., 2004).

Oxidative stress in the form of ROS generation or disruption of the redox balance in the cell is not only involved in cell proliferation and signaling but also induces apoptosis (Venkataramana et al., 2014). In mammalian system, the vital elements involved in apoptosis are *Caspases* and *HSP70*. In the present study, oosporein treatment of cells at 25–100 μM concentrations up-regulated gene expression of apoptosis or oxidative stress inducing proteins and enzymes such as *HSP70*, Cas3, Cas6, and Cas9 mRNA levels in a dose dependent manner. Oosporein induced the gene expression of *HSP70*, Cas3, Cas6, and Cas9 which may lead to apoptosis of studied cells.

To support the *in vitro* cytotoxic effects of oosporein, *in vivo* Balb/C mouse model was used to study the effects of oosporein on target organ systems including kidney and spleen. Histo-pathological examinations clearly evidenced that, there is a significant damage in the target organs in oosporein treated animals compared with sam control treated animals. This further suggests that, oosporein can affect different organ systems systematically and toxic effects are not limited to one organ system. This further suggests that, the need of more research findings to reveal the possible mechanisms of oosporein toxicity to humans and farm animals.

## Conclusion

Present study concludes that the effects of oosporein on MDCK and RAW 264.7 cells is mediated through ROS induced oxidative stress leading to increase in lipid peroxidation, and elevated levels of GSH which in turn results loss in MMP and DNA damage at the cellular level. These events were supported by the q-RT-PCR quantification of the oxidative and apoptotic biomarker gene expression. These experimental results, showed that oosporein may cause strong cytotoxic effects to animals. The high previlance of these fungi in temperate climates further warrants the need of safe food grain storage and processing practies to control the toxic effects of oosporein to humans and live stock.

## Conflict of Interest Statement

The authors declare that the research was conducted in the absence of any commercial or financial relationships that could be construed as a potential conflict of interest.
